# Oleaginous yeast platform for producing biofuels via co-solvent hydrothermal liquefaction

**DOI:** 10.1186/s13068-015-0345-5

**Published:** 2015-10-13

**Authors:** Umakanta Jena, Alex T. McCurdy, Andrew Warren, Hailey Summers, Rhesa N. Ledbetter, S. Kent Hoekman, Lance C. Seefeldt, Jason C. Quinn

**Affiliations:** Desert Research Institute, Reno, NV 89512 USA; Utah State University, Logan, UT 84322 USA

**Keywords:** Biofuel, Hydrothermal liquefaction (HTL), Yeast, Biocrude, Co-solvent, Techno-economic analysis

## Abstract

**Background:**

Oleaginous microorganisms are attractive feedstock for production of liquid biofuels. Direct hydrothermal liquefaction (HTL) is an efficient route that converts whole, wet biomass into an energy-dense liquid fuel precursor, called ‘biocrude’. HTL represents a promising alternative to conventional lipid extraction methods as it does not require a dry feedstock or additional steps for lipid extraction. However, high operating pressure in HTL can pose challenges in reactor sizing and overall operating costs. Through the use of co-solvents the HTL operating pressure can be reduced. The present study investigates low-temperature co-solvent HTL of oleaginous yeast, *Cryptococcus curvatus,* using laboratory batch reactors.

**Results:**

In this study, we report the co-solvent HTL of microbial yeast biomass in an isopropanol–water binary system in the presence or absence of Na_2_CO_3_ catalyst. This novel approach proved to be effective and resulted in significantly higher yield of biocrude (56.4 ± 0.1 %) than that of HTL performed without a co-solvent (49.1 ± 0.4 %)(*p* = 0.001). Addition of Na_2_CO_3_ as a catalyst marginally improved the biocrude yield. The energy content of the resulting biocrude (~37 MJ kg^−1^) was only slightly lower than that of petroleum crude (42 MJ kg^−1^). The HTL process was successful in removing carboxyl groups from fatty acids and creating their associated straight-chain alkanes (C_17_–C_21_). Experimental results were leveraged to inform techno-economic analysis (TEA) of the baseline HTL conversion pathway to evaluate the commercial feasibility of this process. TEA results showed a renewable diesel fuel price of $5.09 per gallon, with the HTL-processing step accounting for approximately 23 % of the total cost for the baseline pathway.

**Conclusions:**

This study shows the feasibility of co-solvent HTL of oleaginous yeast biomass in producing an energy-dense biocrude, and hence provides a platform for adding value to the current dairy industry. Co-solvents can be used to lower the HTL temperature and hence the operating pressure. This process results in a higher biocrude yield at a lower HTL temperature. A conceptual yeast HTL biofuel platform suggests the use of a dairy waste stream for increasing the productivity and sustainability of rural areas while providing a new feedstock (yeast) for generating biofuels.

**Electronic supplementary material:**

The online version of this article (doi:10.1186/s13068-015-0345-5) contains supplementary material, which is available to authorized users.

## Background

Production of fuels and chemicals from sustainable biomass platforms is crucial in addressing the increasing global energy demands and growing concerns of greenhouse gas (GHG) emissions and related adverse climate impacts from the use of fossil fuels. Microbial cells including yeast, algae, and bacteria offer several advantages over the traditional terrestrial feedstocks, including faster biomass production, ability to accumulate lipids, ability to grow in adverse conditions, and scale up feasibility [1–3]. Microbes have been considered as rich sources of lipids that can be converted into biodiesel, renewable diesel, and other liquid hydrocarbon fuels. Oleaginous yeasts are especially promising for biofuel production via lipid extraction and alternative methods, as they can grow on varieties of substrates including molasses, raw materials from the food industry, wastewaters, glycerol, and whey [1, 2, 4]. However, being grown in aqueous media, harvesting and drying are limiting steps for cost-effective conversion of this biomass to fuels [5, 6]. Efficient and economic conversion of yeast to biofuel could improve the biofuel value-chain while addressing energy security needs.

Hydrothermal liquefaction (HTL) is one of the most attractive thermochemical conversion options, in which wet biomass is transformed into a liquid crude (called biocrude) in a single-step process conducted in hot, compressed water [7]. HTL is an attractive option for converting wet biomass because the process itself is conducted in water, reaction rates are enhanced under HTL conditions, recovery of nutrients, and product separations are improved [8]. HTL is preferable over other thermochemical and biochemical conversion approaches, because it is easier to maximize the yields of mid-distillate range bio-oil that can be further processed or co-processed into transport fuels using standard refinery processes [9]. HTL uses the whole biomass (example, yeast) without further drying and pretreatments such as cell lysis and lipid extraction, and it is not necessary to focus on net higher lipid accumulation during the biomass growth. Hence, overall economics in the HTL pathway are more strongly influenced by improvements in biomass productivity rather than extractable lipid content (unlike the solvent-based lipid extraction processes) for production of hydrocarbon fuels [10]. HTL promises higher performance than fast pyrolysis and other thermochemical processes because it can work directly with wet biomass (10–20 % total suspended solids) without the need for additional energy expended for evaporative drying, unlike the other thermochemical processes such as pyrolysis. HTL produced bio-oils have 1.6 times higher energy content, 1.3 times higher carbon and hydrogen, and 0.3 times lower oxygen than fast pyrolysis oil [8]. Previous study by Jena et al. showed HTL had higher oil yields than pyrolysis [11]. Also, hydrocarbon fuel can be produced by hydrotreating HTL oil under relatively milder conditions than oils produced from pyrolysis or lipid extraction processes. Some of the major disadvantages of HTL include the high operating pressure associated with the process (can lead to high capital investments and operational issues), lack of information on standard product separation and purification, and insufficient knowledge about large-scale process development.

Although research on HTL of algae has been widely conducted in recent years [12–14], HTL of yeast has been limited to very few studies [15–17]. Hammerschmidt’s group was one of the first to report HTL of a protein-rich yeast biomass, *Saccharomyces cerevisiae* (baker’s yeast) and showed that it was possible to produce biocrude at process temperatures over 400 °C. Valdez et al. [16] performed HTL of yeast at 350 °C and demonstrated that a faster heating rate could result in ~48 % biocrude from *S. cerevisiae* corresponding to a chemical energy recovery efficiency of ~63 %. Miao et al. [15] reported a two-step sequential hydrothermal fractionation of *Cryptococus curvatus* (referred to as sequential HTL, or SEQHTL by the authors), which isolated value-added co-products in the first step and biocrude in the subsequent HTL of the residual biomass. Research has shown the feasibility of the thermochemical conversion of yeast biomass through HTL; however, there has been minimal work dedicated to decreasing the operational pressure and temperature of the conversion process.

Despite growing interests in HTL research in academic institutions and industries around the world, commercialization of this technology is not presently realistic [9]. Further research is needed to address several challenges and technical barriers such as process optimization, reactor development, and cost. Available literature reviews reveal that product yields in HTL are largely affected by the operating conditions including temperature, pressure, residence time, type and composition of feedstock, presence of catalysts, and use of reducing gas/hydrogen donors [18–20]. Among all these parameters, HTL product distribution and biocrude yield are most sensitive to process temperature. A temperature of 350 °C has been reported to be optimum for biocrude yield [7, 19, 21]. The corresponding working pressure is 18–20 MPa (2650–2900 psi), which can lead to high capital and operating costs due to the required high pressure vessels, valves, and other system components [9]. Operating temperature is a key parameter that dominates the process energetics of any thermochemical process, including HTL. Simple thermodynamic calculations reveal that an HTL process when operated with 10 % solids content in the slurry at 350 °C requires approximately 1290 MJ m^−3^ of sensible heat, compared to ~1100 and ~890 MJ m^−3^ at 300, and 250 °C, respectively. There is a need to lower energy and capital cost requirements if the process is to be economically sustainable. Binary solvents (water–alcohol mixture) and catalysts can reduce the HTL operating temperature significantly. Unique thermodynamic properties (Gibbs free energy, enthalpy, entropy, etc.) of binary solvents allow certain reactions to proceed at a faster rate than is possible in a single solvent (example, water). Addition of alcohols (such as methanol, ethanol, and propanol) lowers the dielectric constant of the solvent mixture and significantly lowers its critical values (temperature and pressure) as well as reduces oxygen content of the product biocrude [22–24]. Recently, researchers from around the world have reported the use of water–alcohol binary solvents in HTL of biomass [22–28]. Water–alcohol reaction systems have several advantages including significantly lower critical temperature (*T*_c_) and critical pressure (*P*_c_) than pure water, ability to act as an active hydrogen donor in HTL, ability to dissolve macromolecules in the feedstock, ability to reduce net oxygen content of product biocrude, and ability to react with acidic components to form fatty acid esters [22, 26, 28]. Studies using alcohol co-solvents (ethanol, propanol) have been reported for HTL treatment of lignocellulosic feedstocks [22, 25, 27] and algal biomass [24, 28]. In these studies, synergistic effects of water–ethanol not only improved the biocrude yield, but were performed at lower process temperatures. We chose to use isopropanol in the present study because water–isopropanol binary mixture has a lower autogenous pressure than the water–ethanol and water–methanol systems at the same temperature [22]. Hence, using isopropanol–water binary solvent in HTL, one can achieve the same or higher yield of biocrude even at a lower reaction severity (temperature and pressure) than that of other lower alcohol–water systems (example, ethanol–water) and hence, can reduce the costs of pressure vessels and pipelines. Use of catalysts in HTL is also known to significantly affect the product distribution [20, 29]. Among different catalysts used in HTL, alkali (sodium and potassium) materials have been found to be the most promising [21, 29]. Jena et al. [29] reported increased oil yields of 20–30 % at 300–350 °C due to addition of 5 % Na_2_CO_3_ in the HTL of *S. platensis*. In addition, Na_2_CO_3_ is inexpensive, non-toxic, and readily available.

This study reports the results from the investigation of co-solvent HTL of oleaginous yeast, *C. curvatus,* using a water–isopropanol binary solvent. The yeast species *C. curvatus* are known to grow well and accumulate high amounts of lipids using waste generated from the dairy industry. The novelty of the study lies in the co-solvent use and in the integration of baseline HTL experimental data with a techno-economic analysis to understand the potential impact that HTL can have on a systems level analysis.

## Results and discussion

### Yeast biomass characterization

Yeast growth on simulated delactosed permeate medium (at 40 g L^−1^ lactose concentration) resulted in biomass concentration of 21.3–34.2 g L^−1^ (dry wt.) when supplied with a nitrogen source (0.25–5.0 g L^−1^ ammonium sulfate) (data not shown). The above results were obtained at an optimal substrate (lactose) at fourfold dilution. Based on experimental results the biomass yield was estimated as 53.2–85.5 %. Resulting biomass reported 28–40 % lipids fraction. Further information on yeast growth can be found elsewhere [30]. The elemental, proximate, and biochemical composition of yeast biomass, *C. curvatus* used in the present study are presented in Table 1. The *C. curvatus* biomass had large amounts of organic matter (~90 % volatiles and ~2 % fixed carbon) and ~8 % ash content. Higher organic content, lower ash content, and higher energy content make it an excellent feedstock for biofuel conversion compared to other biomass feedstocks such as lignocellulosic feedstocks, sewage sludge, animal manure, and municipal solid wastes [14, 31, 32]. The HHV of yeast feedstock used in this study was 24.88 MJ kg^−1^ (Table 1), which is higher than that of many lignocellulosic and algae feedstocks, which are typically 12–20 MJ kg^−1^. Also, the organic constituents (lipids, protein, and carbohydrates) of the yeast are at similar or higher levels than most species of algae that have been widely used in HTL [7, 21, 33]. Large amounts of lipids (32.8 %) and small amounts of proteins (16.0 %) make it a promising feedstock for biofuel conversion. The fatty acid analyses (Table 1) show large amounts of long chain fatty acids (C16 and C18) with about 40 % of them being unsaturated.

**Table 1:** Composition of yeast biomass samples (as received basis wt %)

Ultimate analysis (elemental)		Proximate analysis	
C, %	54.92 ± 0.79	Moisture, %	3.54 ± 0.27
H, %	8.73 ± 0.20	Volatiles, %	90.14 ± 2.04
N, %	2.40 ± 0.14	Fixed carbon, %	2.50 ± 0.50
O^c^, %	33.85	Ash, %	7.82 ± 0.20
Chemical content		Fatty acids composition, % of total fatty acids^a,b^	
Carbohydrates, %	19.40 ± 0.15	C14	0
Lipids, %	32.77 ± 1.29	C15	0
Crude protein, %	16.00 ± 0.38	C16	15.5
		C17	0
Energy content		C18	83.4
HHV, MJ kg^−1^	24.88 ± 0.53	C20+	1.1

*HHV* higher heating value

^a^Values are relative percent (%) of the transesterifiable lipids calculated by the use of in situ transesterification and are presented relative to the area under the peak from GC/MS analysis of the transesterifiable lipid portion ranging from retention time of 5–15 min

^b^About 34 % of total fatty acids were mono-unsaturated and 5.6 % of total fatty acids are poly-unsaturated

^c^By difference

### HTL product distribution and mass balance

Product yields (recovery) in HTL from different treatment conditions are presented in Table 2. Between 86 and 92 % of the mass was recovered in the forms of biocrude, solids (char), gases, and non-volatile residues (NVR). Mass loss of 8–14 % could be attributed to water-soluble organics (that were not analyzed in this study), unmeasured gaseous products (in the case of 2-chamber runs), produced water, and experimental errors. Carbon mass closure could not be conducted in the present study, as ultimate analyses of the water-soluble products and some of the char products were not performed. However, carbon–hydrogen recovery in the biocrude (the ratio of the carbon and hydrogen recovered in the biocrude to the carbon and hydrogen present in the feedstock) was determined, and found to be in the range of 68.2–77.3 %. Addition of catalyst increased the carbon–hydrogen recovery in the biocrude by 6.7–8.9 %. Under all process conditions, HTL of yeast resulted in 49.1–57.9 % of total biocrude yield (B1 + B2; see “Methods” section), which was much higher than the starting lipid content (~33.0 %) of the feedstock. This indicates that HTL can transform most of the organics (including lipids) present in the yeast biomass into a liquid biocrude product. In HTL, the biomass macromolecules (lipids, proteins, and carbohydrates) breakdown into corresponding monomers via hydrolysis/solvolysis, which further decompose into various intermediates [8, 24, 34, 35]. Reactions of monomers and intermediates (decarboxylation, deamination, and condensation) lead to formation of liquids, gases, and solid products. For example, breakdown of lipids, proteins, and carbohydrates in yeast result in amino acids, triglycerides, and sugar molecules that end up in forming amides, amines, acid and esters (as discussed in the “GC–MS results”). The biocrude B1 fraction (obtained from DCM-assisted separation) was visibly lighter than the B2 fraction (obtained from acetone-assisted separation) although both fractions were dark brown in color and had a characteristic smoky odor. The B1 fraction represented 70–75 % of the total biocrude yield. The distribution of B1 and B2 biocrude fractions are presented in Additional file 1: Figure S1.

**Table 2:** Experimental results from HTL of yeast biomass; biocrude yield, properties, and product and energy recovery

	Non-catalytic HTL, w/o co-solvent	Catalytic HTL^a^, w/o co-solvent	Non-catalytic HTL, with co-solvent^b^	Catalytic HTL^a^, with co-solvent
Temperature/reactor type	300 °C, 2-L	300 °C, 2-L	240 °C, 2-chamber	240 °C, 2-chamber
Mass balance				
Biocrude recovery^c^, %	49.11	52.61	56.38	57.94
Solids recovery, %	20.06	21.35	30.65	32.83
Gaseous recovery, %	1.09	1.18	nd	nd
NVR recovery, %	16.28	10.34	1.46	1.26
Total mass recovery, %	86.54	85.48	88.49	92.03
Mass lost, %	13.46	14.52	11.51	7.97
Biocrude composition and properties				
C, %	77.54	77.68	74.67	73.19
H, %	11.51	11.38	12.56	11.68
N, %	1.96	2.19	0.51	1.79
O^d^, %	12.73	12.91	12.58	14.32
O^e^/C	0.12	0.12	0.13	0.15
N^e^/C	0.02	0.02	0.01	0.02
Ash, %	0.06	0.03	0.05	0.04
HHV, MJ kg^−1^	36.55	37.00	36.65	37.01
ER_biocrude_, %	72.14	78.25	83.05	86.20

All HTL runs were performed for 30 min residence time. In case of catalytic runs the initial mass of catalysts were not included for the % mass lost calculation

*HHV* higher heating value, *ER*_*biocrude*_ energy recovery in biocrude

^a^Catalyst was Na_2_CO_3_ (5 % (w/w) of feedstock)

^b^Co-solvent was isopropanol (1:1, in water)

^c^Biocrude yield was the combined mass of DCM soluble and acetone soluble compounds in Fig. 7

^d^By difference

^e^Atomic ratio

The yield of biocrude from yeast obtained in this study was significantly higher than from many other previously studied feedstocks including microalgae at similar HTL operating temperature and reaction time [7, 21]. The biocrude yield was also higher than reported by Hammerschmidt et al. [17] for the yeast, *S. cerevisiae*. The difference in yield between Hammerschmidt et al. [17] and the present study could be attributed to differences in feedstock composition (this study used high-lipid yeast compared to high-protein yeast in the study in comparison), reaction parameters (residence time, temperature, catalysts, co-solvent) and reactor types. Moreover, severe reaction conditions (at 400 °C) in the study by Hammerschmidt et al. [17] favored hydrothermal gasification (supercritical gasification), while our reaction conditions remained within the HTL regime (<350 °C) favoring biocrude production. Our yield results are more similar to the results obtained by Valdez et al. [16] and Miao et al. [15]. Biocrude yield reported by Valdez et al. [16] from *S. cerevisiae* was ~40 % for HTL conducted at 350 °C, which increased to ~50 % with fast HTL (that was conducted at a higher heating rate, ~276 °C min^−1^). Heating rate is known to affect reaction kinetics significantly in thermal conversion processes, enhancing the pyrolytic and hydrolytic reactions in HTL [36]. Hence one would expect higher oil yield at a higher heating rate. In the present study, even at lower temperature and lower heating rates (300 °C, 3–5.5 °C min^−1^), we obtained 49.1 % biocrude yield. This could be due to the higher lipids (32.8 %) in the yeast feedstock compared to 2.7 % lipids in the study by Valdez et al. [16].

Addition of catalyst and co-solvent significantly increased biocrude yield. Jena et al. [29] reported significantly higher biocrude yield in catalytic HTL of algae using Na_2_CO_3_. Reactions of sodium carbonate and water with carbon monoxide eventually generate hydroxyl (OH^−^) and formate (HCOO^−^) ions in the multi-step process that catalyzes oil-production [31]. The ANOVA test on the biocrude yields showed significant difference in yields between various treatments (*p* = 0.001, *F* = 49.13). Co-solvent HTL, using an isopropanol–water binary solvent, resulted in 56.4 % biocrude in the present study. This binary solvent has lower critical temperature (T) and pressure (P) (~311.3 °C and 10.2 MPa or ~1484 psi) than water alone (374.1 °C and 22.1 MPa or ~3200 psi) [22]. Under the reaction conditions used in this study, the binary solvent approaches the supercritical state, and thus is a highly reactive medium for HTL reactions that favor increased biocrude yield. Isopropanol is believed to enhance hydrogen donation and hydoxyalkylation, which helps in alcoholysis and other HTL reactions [36]. There was an additional 7.1 % increase in biocrude yield due to addition of Na_2_CO_3_ in the HTL without co-solvent; however, the catalyst did not significantly affect the biocrude yield in co-solvent HTL experiments in the present study. Miao et al. [15] reported biocrude yields of ~58 and ~56 % at 240 and 300 °C, respectively, even without the use of co-solvent and catalyst. However, no information was provided on the feedstock composition (lipids, proteins, carbohydrates) and hence, direct comparison of results with the above study may not be appropriate.

#### Molecular characterization of biocrude

##### Elemental composition and HHV

The elemental composition and HHV of biocrudes obtained from different HTL treatments are presented in Table 2. HTL resulted in significant reduction of oxygen (57–63 % less than the starting feedstock) and higher elemental C and H. Elemental C was ~78 % for biocrude obtained at 300 °C and ~74 % for biocrude obtained at 240 °C (for both non-catalytic and catalytic runs). These values were considerably higher than the ~55 % carbon content of yeast feedstock. Lower O:C and N:C ratios resulted in higher energy content (MJ kg^−1^). The energy content of yeast-derived biocrude from HTL was ~37 MJ kg^−1^, compared to ~43 MJ kg^−1^ for diesel fuel, and is comparable to other biocrudes obtained from yeasts [15, 16] and algae feedstocks [29, 33]. Low O, N, and ash values suggest that biocrude from HTL of yeast could be readily upgraded into refinery-grade fuels (or intermediates) by catalytic hydrogenation or deoxygenation methods and other thermal treatments [37, 38].

##### GC–MS results

**Fig. 1:**
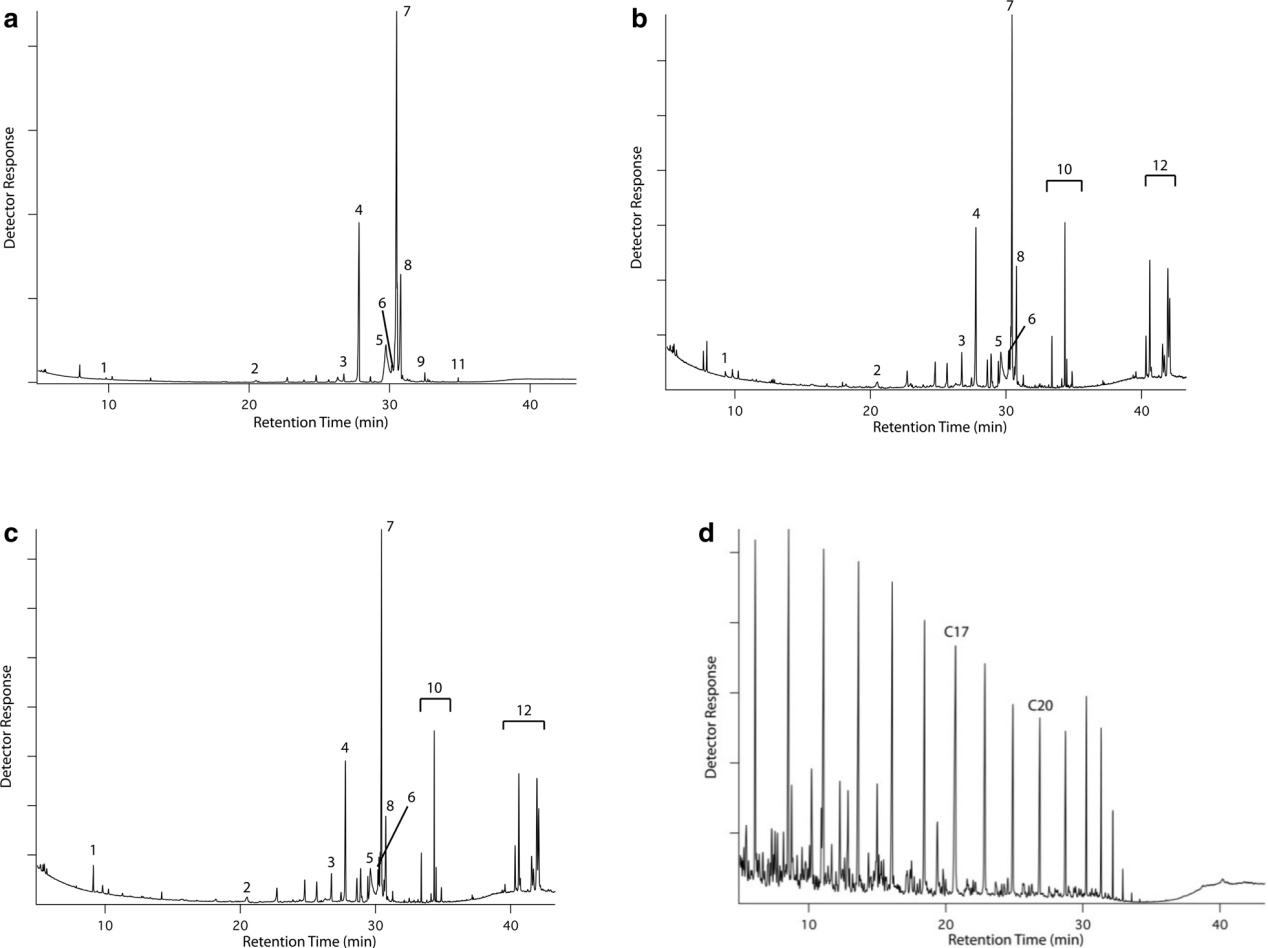
GC–MS chromatograms of DCM-extracted biocrudes. **a** Non-catalytic HTL without co-solvent, 300 °C; **b** non-catalytic HTL with co-solvent, 240 °C; **c** catalytic HTL with co-solvent, 240 °C; and **d** reference diesel fuel; [peaks identified- *1* glycerol; *2* heptadecane; *3* eicosane, *4* palmitic acid (TMSE), *5* oleic acid (non-derivative), *6* stearic acid (non-derivative), *7* oleic acid (TMSE), *8* stearic acid (TMSE), *9* hexadecenamide, *10* monoglycerides (TMSE), *11* tetracosanoic acid (TMSE), *12* diglycerides (TMSE); triglyceride peaks are not shown]; TMSE: trimethylsilyl ester and trimethylsilyl ether

Figure 1a–c depicts the GC–MS characterization of the biocrude samples resulting from various HTL treatment conditions with Fig. 1d showing the GC–MS of a reference diesel fuel. Triglycerides (TAG) could not be resolved with the mass spectrometer and were quantified by FID detector as detailed in the “Methods” section. GC–MS shows a wide range of higher fatty acids (palmitic acid, stearic acid, oleic acid), straight-chain alkanes (C_17_–C_21_), amides, glycerides, and glycerol. Most of these compounds were derived from the triglycerides, which contribute to the higher lipid content (~33 %) of the starting feedstock. Amides likely arise from reactions of proteins into amino acids, and subsequently to amides at higher temperature HTL treatments. Percentage abundance of different compounds is summarized in Additional file 1: Table S1. Composition of both B1 and B2 biocrudes obtained from DCM and acetone-assisted solvent separation, respectively, were similar although there were minor differences in the relative abundance of certain compounds. Generally, biocrude from HTL conducted at 300 °C contained a higher abundance of derivatized fatty acids than biocrude obtained at 240 °C, which showed higher abundance of glycerides (mono-, di-, and tri-), and derivatized glycerol (Additional file 1: Table S1). Relative abundance of compounds suggests that hydrolysis is a major reaction in the case of lower temperature HTL conducted at 240 °C (either with or without co-solvent isopropanol). Compressed hydrothermal medium (water or water–alcohol mixture) leads to hydrolysis of triglycerides in the yeast biomass to form di-, and mono-glycerides along with fatty acid derivatives and glycerol [39, 40]. The hydrolysis reaction occurs in three stepwise homogenous first-order reversible reactions [39] as shown in Eqs. (1–3): (1) triglyceride (TG) is hydrolyzed to diglycerides (DG), (2) DG is hydrolyzed to monoglycerides (MG), and (3) MG is hydrolyzed to glycerol. Each step involves production of a fatty acid, with free glycerol being formed in the final step.

1 $$ \begin{array}{*{20}c} {{\text{C}}_{ 3} {\text{H}}_{ 5} .\left( {\text{OOCR}} \right)_{3}  } \\ {\text{triglyceride}} \\ \end{array} \begin{array}{*{20}c} + \\ {} \\ \end{array} \begin{array}{*{20}c} {{\text{H}}_{ 2} {\text{O}}} \\ {\text{water}} \\ \end{array} \begin{array}{*{20}c} \leftrightarrow \\ {} \\ \end{array} \begin{array}{*{20}c} {{\text{C}}_{ 3} {\text{H}}_{ 5} \left( {\text{OH}} \right)} \\ {} \\ \end{array} \begin{array}{*{20}c} \cdot \\ {} \\ \end{array} \begin{array}{*{20}c} {({\text{OOCR}})_{2} } \\ {\text{diglyceride}} \\ \end{array} \begin{array}{*{20}c} + \\ {} \\ \end{array} \begin{array}{*{20}c} { {\text{RCOOH}}} \\ {\text{fatty acid}} \\ \end{array} $$

2 $$ \begin{array}{*{20}c} {{\text{C}}_{ 3} {\text{H}}_{ 5} \left( {\text{OH}} \right).\left( {\text{OOCR}} \right)_{ 2}  } \\ {\text{diglyceride}} \\ \end{array} \begin{array}{*{20}c} + \\ {} \\ \end{array} \begin{array}{*{20}c} {{\text{H}}_{ 2} {\text{O}}} \\ {\text{water}} \\ \end{array} \begin{array}{*{20}c} \leftrightarrow \\ {} \\ \end{array} \begin{array}{*{20}c} {{\text{C}}_{ 3} {\text{H}}_{ 5} \left( {\text{OH}} \right)_{ 2} } \\ {} \\ \end{array} \begin{array}{*{20}c} \cdot \\ {} \\ \end{array} \begin{array}{*{20}c} {({\text{OOCR}}) } \\ {\text{monoglyceride}} \\ \end{array} \begin{array}{*{20}c} + \\ {} \\ \end{array} \begin{array}{*{20}c} { {\text{RCOOH}}} \\ {\text{fatty acid}} \\ \end{array} $$

3 $$ \begin{array}{*{20}c} {{{\text{C}}_{ 3} {\text{H}}_{ 5} \left( {\text{OH}} \right)_{ 2} }.\left( {\text{OOCR}} \right)} \\ {\text{monoglyceride}} \\ \end{array} \begin{array}{*{20}c} + \\ {} \\ \end{array} \begin{array}{*{20}c} {{\text{H}}_{ 2} {\text{O}}} \\ {\text{water}} \\ \end{array} \begin{array}{*{20}c} \leftrightarrow \\ {} \\ \end{array} \begin{array}{*{20}c} {{\text{C}}_{ 3} {\text{H}}_{ 5} \left( {\text{OH}} \right)_{ 3} } \\ {\text{glycerol}} \\  \end{array} \begin{array}{*{20}c} + \\ {} \\ \end{array} \begin{array}{*{20}c} { {\text{RCOOH}}} \\ {\text{fatty acid}} \\ \end{array} $$

**Fig. 2:**
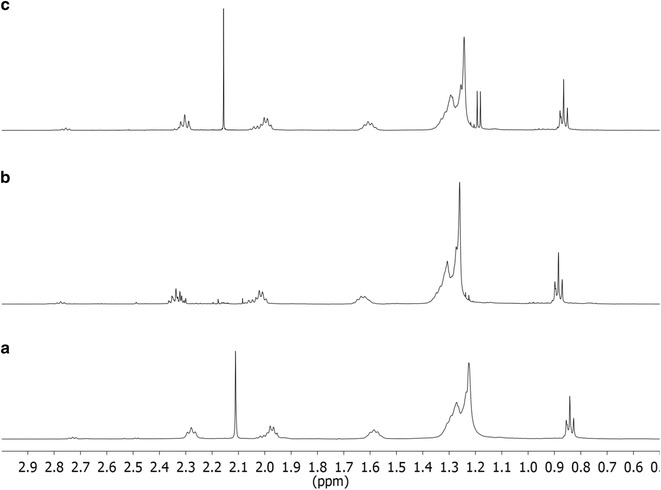
Expanded (0–3 ppm) ^1^H NMR spectra (500 MHz, 25 °C, CDCl_3_) of biocrudes. **a** HTL run, 2-L Parr reactor, at 300 °C, w/o co-solvent, with Na_2_CO_3_ catalyst, **b** non-catalytic HTL runs, 2-chamber reactor, 240 °C, with co-solvent, **c** catalytic HTL runs, 2-chamber reactor, 240 °C, with co-solvent; the *sharp peaks* near 2.1 ppm (seen in spectra “**a**” and “**c**”) is due to acetone impurity (full NMR spectra are presented in supplementary information, Additional file 1: Figure S2)

For biocrude samples obtained from co-solvent HTL, small peaks for isopropyl esters were observed between the larger peaks for oleic acid (TMSE) and stearic acid (TMSE). However, their abundances were too small to be shown in the GC–MS chromatograms of Fig. 1. The presence of isopropyl esters was confirmed by the NMR spectra (Fig. 2).

##### NMR analysis

^1^H NMR spectroscopy provides information about the types and amounts of functional groups in the biocrude molecules. Identity of a functional group is determined from the chemical shift, whereas its relative abundance is estimated from the peak area. Figure 2 shows the 0–3 ppm chemical shift region of the ^1^H NMR spectra of biocrudes obtained from HTL of yeast with and without co-solvent (full NMR spectra are presented in the Additional file 1: Figure S2). Most of the strong peaks reside within the 0.8–2.3 ppm range, where aliphatic methyl and methylene protons appear. The peaks at 0.88 and 1.25 ppm are characteristics of terminal methyl and methylene groups in alkyl chains, respectively [41]. Appearance of these strong peaks is in good agreement with the abundance of fatty acids and alkanes as shown by GC–MS (Fig. 1). The appearance of peaks at about 1.35–1.64 and 2.00–2.35 ppm are expected from protons on carbon atoms β and α, respectively, to an acyl group, as in a fatty acid. Biocrude samples obtained from co-solvent HTL experiments showed a number of peaks at 3.65–4.33 ppm (Additional file 1: Figure S2), some of which may be attributed to methine protons of isopropyl esters. These samples also showed doublets near 1.2 ppm, which are attributed to the methyl groups within isopropyl esters.

##### FT-IR analysis

**Fig. 3:**
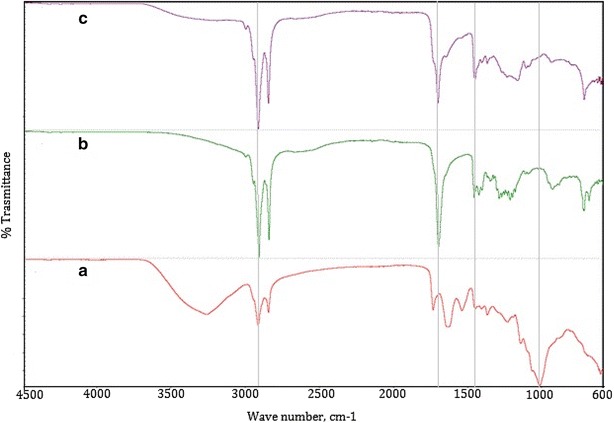
FTIR of yeast raw material (**a**) and biocrudes obtained from non-catalytic HTL without co-solvent (**b**) and with co-solvent (**c**)

FT-IR spectra of raw material (yeast biomass) and biocrudes from HTL at two different conditions are shown in Fig. 3. It can be seen that the spectra of biocrudes obtained from HTL without and with isopropanol co-solvent (Fig. 3b, c, respectively) are similar. The broad vibration at around 3300 cm^−1^ in spectrum a of Fig. 3 can be attributed to O–H stretching vibrations indicating the presence of polysaccharides and proteins in yeast raw material. The stretching vibration in spectrum c (at 3400 cm^−1^) of the biocrude-obtained co-solvent HTL is attributed to the presence of alcohols and phenols, which was not very prominent in the spectrum b (for biocrude obtained in non-catalytic HTL without any co-solvent). The higher resolution and strong absorption peaks from 2965 to 2855 cm^−1^ in all spectra are attributed to C–H stretching vibrations of CH_3_ and CH_2_. The strong, broad band near 1000 cm^−1^ in the raw yeast spectrum, which is attributed to C–O stretching in carbohydrates and triglycerides, largely disappears in the biocrude spectra. This is evidence of hydrolysis occurring in the HTL process, breaking apart C–O–C linkages and producing smaller molecules with more carbonyl groups (ketones and organic acids). The strong bands near 1750 cm^−1^ in the biocrude spectra are attributed to these carbonyl groups. The greater complexity of the carbonyl band in the spectrum of the biocrude from co-solvent HTL may be due to formation of isopropyl esters from reaction of isopropanol with fatty acids.

#### Analyses of solids

**Fig. 4:**
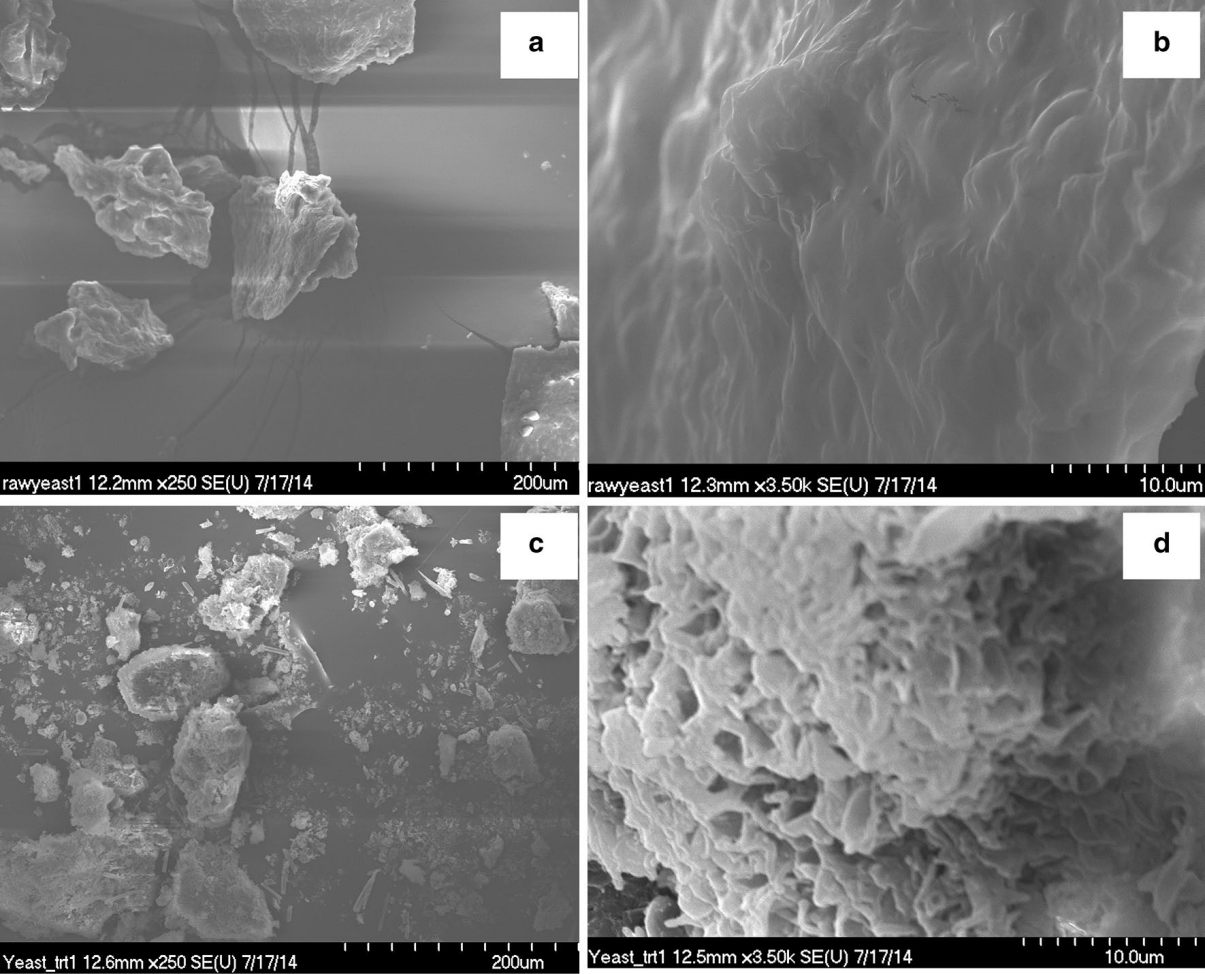
Scanning electron microscope (SEM) images. **a**, **b** for raw (unprocessed) yeast, and c-d for solid char obtained from non-catalytic co-solvent HTL at 240 °C. Left column SEM images **a**, **c** were obtained at ×250 magnification; SEM images **b**, **d** were obtained at ×3500 magnifications

HTL of yeast resulted in 20–33 % solid chars (Table 2). HTL conducted at 300 °C resulted in lower solids than at 240 °C, which is in good agreement with studies using algae [7] and other feedstocks [22, 25, 27]. Higher HTL temperature favors the conversion of hydrolyzed solids into oil-like compounds and gases. The solid chars obtained in this study contained large amounts of volatile matter (84–87 %) and small amounts of fixed carbon (~8.5 %), Additional file 1: Table S2. This is quite different from solid chars that are produced from many other biomass feedstocks including wood and algae. Non-catalytic experiments conducted at 300 °C resulted in char with lower ash percentage than did experiments with co-solvent HTL at 240 °C. At all temperature conditions, HTL-solids had lower ash than that of the starting biomass, suggesting that some ash constituents were removed in the aqueous product stream. The solid char also had higher elemental C (68–69 %), H (9–11 %), and heating value (28–31 MJ kg^−1^) than the raw yeast, suggesting that this product can be further used as an energy-dense fuel or feedstock. Morphological changes in solids due to HTL were studied using SEM. Figure 4a–d shows SEM micrographs of the unprocessed (raw) yeast and solid char from co-solvent, non-catalytic HTL treatment at 240 °C. The unprocessed yeast feedstock had distinct solid particles and connected surfaces, whereas the solid char displayed disintegrated particles with splintered surfaces. A closer micrograph (at 3500× magnification) showed a distinct change in morphology between the raw yeast and the HTL-obtained solid char (Fig. 4b, d). The unprocessed feedstock showed an intact (unbroken) surface, whereas the solid char displayed numerous visible cracks and open pores on the surface, suggesting that deformation occurred due to HTL treatment.

#### Analyses of gases

Gaseous products could be collected, quantified, and identified only from the experiments conducted in the 2-L Parr reactor system. When using the 2-chamber reactor system, gases were simply vented to the atmosphere after cooling the reactor. The normalized gaseous products from the 2-L Parr reactor system consisted primarily of CO_2_ (>93 %), small amounts of CO, and trace amounts of hydrocarbon gases (CH_4_, C_2_H_6_) (Additional file 1: Figure S3). CO and CO_2_ yields represented about 0.03 and 1.06 %, respectively, of the starting yeast mass in the non-catalytic HTL experiment conducted at 300 °C and 30 min residence time. The formation of CO, CO_2_, and hydrocarbon gases can be explained by a combination of hydrothermal reactions such as decarboxylation, water gas shift reaction, methane forming reaction, and gasification of solid residues (char), which are favored by highly reactive hot compressed water [7]. Addition of Na_2_CO_3_ increased the CO and CO_2_ yield by 30.8 and 8.2 %, respectively. Na_2_CO_3_ has been reported to catalyze the conversion processes that increase gaseous products from different types of biomass including algae [21, 29] and woody biomass [31]. Higher concentrations of CO_2_ in the presence of Na_2_CO_3_ can be explained by the reaction of sodium carbonate and water with carbon monoxide (Eq. 4) [31], which eventually generates hydroxyl (OH^−^) and formate (HCOO^−^) ions that catalyze the production of biocrude.

4 $$ {\text{Na}}_{2} {\text{CO}}_{3}   + 2{\text{CO}} + {\text{H}}_{2} {\text{O}} \leftrightarrow 2 {\text{HCOONa}}  + {\text{CO}}_{2} $$

Our gaseous composition results from both catalytic and non-catalytic HTL are similar to those from previous studies on algae [29].

#### Analyses of aqueous co-phase products (ACP)

The ACP generated from HTL of yeast was characterized by light brown color, mild smoky odor, and acidic pH (3.7–4.1) (Table 3). The acidic pH of this phase can be explained by large amounts of carboxylic acids, fatty acids, and amino acids resulting from hydrothermal reactions in the sub-critical reaction environment. Water-soluble products in ACP were analyzed for N, P, and TOC; the results are presented in Table 3. The ACP was characterized by the presence of large amounts of dissolved NH_3_-N (438–659 mg L^−1^), Kjeldahl-N (KN) (800–1220 mg L^−1^) and total P (1254–1794 mg L^−1^). Small amounts of NO_3_-N were also found (0.10–22 mg L^−1^) in the ACP. The N recovery in the ACP represented 15–21 % of total biomass-N, of which 55–71 % constituted NH_3_-N. Higher N-recoveries were observed at higher HTL temperature (300 °C). Use of catalyst (Na_2_CO_3_) resulted in a decrease in the dissolved KN, whereas use of co-solvent HTL increased the total P in the ACP. Owing to N and P disposition and recovery in ACP, recycling of water-soluble nutrients (N and P) is possible. Recycling suitable amounts of HTL-processed ACP has demonstrated significant growth of algae biomass and lipid production in previous studies [21, 42–44]. Espinosa-Gonzalez et al. [45] recently reported growing oleaginous yeast, *C. curvatus*, using aqueous fractions derived from hydrothermal processing of the yeast.

**Table 3:** Analysis of water-soluble aqueous phase co-products (ACP)

HTL experimental conditions and reactor type	N and P, expressed in mg L^−1^					NVR, %	TOC, mg L^−1^
	NO_3_ and NO_2_	NH_3_–N	KN	Total P	pH		
Non-catalytic, 300 °C, 2-L Parr	0.10	659	1220	1254	3.74	16.28	4281
Catalytic, 300 °C, 2-L Parr	0.24	502	979	1335	3.92	10.34	4611
Non-catalytic, co-solvent 240 °C, 2-chamber	0.20	521	927	1722	3.83	1.46	5091
Catalytic, co-solvent 240 °C, 2-chamber	0.22	438	800	1794	4.11	1.26	5643

All HTL runs were performed for 30 min residence time

*N* nitrogen, *KN* Kjeldahl nitrogen, *NVR* non-volatile residue, *TP* total phosphorous, *TOC* total organic carbon

Total organic carbon (TOC) is an indirect measure of organic molecules present in process effluents or wastewater. TOC measurements of the ACP can determine the fate of the process stream, i.e., whether it can be used in further growth of plants and microorganisms, or it is suitable for discharge into water bodies. In the present study, TOC concentrations of ACP were 4281–5643 mg L^−1^ (Table 3) which represents 4.7–6.2 % of the total starting biomass. HTL conducted at low-temperature co-solvent runs resulted in 20–30 % higher TOC than the higher temperature treatments. These high TOC concentrations may pose discharge issues due to stringent environmental regulations, and may require further treatments. Based on literature reports, the dissolved organics contain sugars, organic acids, and traces of amine/aromatic compounds and can be converted into methane-rich gases by hydrothermal gasification [46].

### Energy balance in HTL

Chemical energy balance with respect to the starting yeast feedstock is presented in Additional file 1: Table S2. In any closed process, total chemical energy entering the process should equal the chemical energy transformed in the various products. In case of HTL experiments conducted in a 2-L Parr reactor system, the gaseous products could be captured and measured. The total chemical energy in the major products (biocrude and solid char) was over 96 % in the non-catalytic HTL run at 300 °C. For the co-solvent HTL treatments, the total chemical energy in products was higher than the chemical energy in the starting feedstock (2488 MJ). The gain in chemical energy in products (Additional file 1: Table S2) is due in part to volatile compounds adding to the solids by hydrolysis of glycerides to form carboxylic acids and glycerol [39, 40] as per Eqs. (1–3) described above. In addition, a small amount of mass (and hence energy) from the co-solvent was incorporated into the products in the form of isopropyl esters as indicated by the GC–MS and ^1^H NMR results discussed above.

The chemical energy recovery in the biocrude (ER_biocrude_) was 72–86 % for the HTL of yeast. The use of co-solvent, isopropanol, significantly increased the ER_biocrude_. ER_biocrude_ for the catalytic HTL using Na_2_CO_3_ was higher than that of non-catalytic runs at both temperature conditions (240 and 300 °C) (data were analyzed at 0.05 significance level). Jena et al. [29] also reported that Na_2_CO_3_ resulted in significant increase in ER_biocrude_ values for HTL of microalgae, *S. platensis*. It is important to note that the chemical energy present in the co-solvent (isopropanol) is not taken into account as the total solvent recovered in the process could not be accurately determined and analyzed. For a commercial application, it would be important to conduct an overall material and energy balance with the co-solvent HTL to determine the possibility of solvent recycling and reuse.

### Economic evaluation

**Fig. 5:**
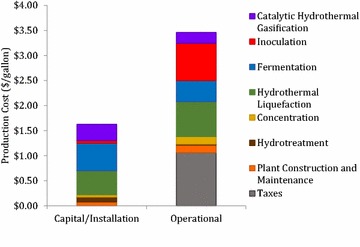
Preliminary economic results of biofuel production via yeast platform. In the studied platform of yeast fermentation on whey permeate is followed by HTL processing; the costs are separated into operational and capital costs

Results from biological and thermochemical conversion experimentation were incorporated into an engineering system model to evaluate the techno-economic feasibility of biofuel production through HTL conversion with and without catalyst based on the methods presented in Summers et al. [30]. The primary function of the model is to track mass and energy requirements for each process at a simulated annual production level of 72 million liters (19 million gallons) of drop-in biofuel. Cost data were gathered to account for the startup capital, installation, and operational expenses of a proposed industrial-scale production plant. The baseline HTL-based production pathways were analyzed, utilizing experimental results summarized in Table 2. For the HTL cases without co-solvent, results show that inclusion of the catalyst reduced the conversion costs from $5.09 per gallon of biocrude to $4.78. This reduced cost is primarily due to improved biocrude yield caused by the catalyst, despite a small additional cost associated with the catalyst. Operational and capital costs are presented on a process level in Fig. 5 for the baseline HTL process.

Results from the sensitivity analysis discussed in the “Methods” section indicated that biomass yields, HTL conversion efficiency, and the fermentation system are the most sensitive inputs to the overall production cost of biofuel. Biomass yields and HTL conversion efficiencies were experimentally optimized in this study; however, fermenter energetics and economics were only moderately investigated. Detailed results integrating sensitivity to fermentation is presented in Summers et al. [30]. The fermentation costs account for approximately 35 % of the total baseline production costs, suggesting a potential for optimization of the fermenters to reduce the overall cost of biofuel. Results show the HTL system represents 9.4 % of the total capital and 13.8 % of the total operational costs, highlighting the need to decrease the operating temperature and pressure. Experimental work was conducted with co-solvents to explore the feasibility of decreasing the operational pressure and temperature and determine the corresponding impacts on yield. For the HTL cases with co-solvent, experimental data were not sufficient to support a large-scale TEA assessment. It is expected that with reasonable co-solvent recovery, the economics would be improved based on the decrease in operational and capital costs associated with the HTL system. Experimental work focused on the optimization of co-solvent volumes required for biocrude recovery, as well as co-solvent recovery during conversion, has the potential to reduce the minimum selling price of the HTL-derived fuel. Finally, inclusion of co-product pathways for use of residual biomass as feed or fertilizer could further reduce the biofuel selling point, although this was not investigated in the present study. With the inclusion of fermentation cost reduction efforts, co-solvent optimization, and co-product stream utilization, this HTL-based conversion pathway has the potential to be economically competitive with traditional biofuel production pathways.

### Conceptual yeast HTL platform for producing biofuels from delactosed permeate dairy byproducts

**Fig. 6:**
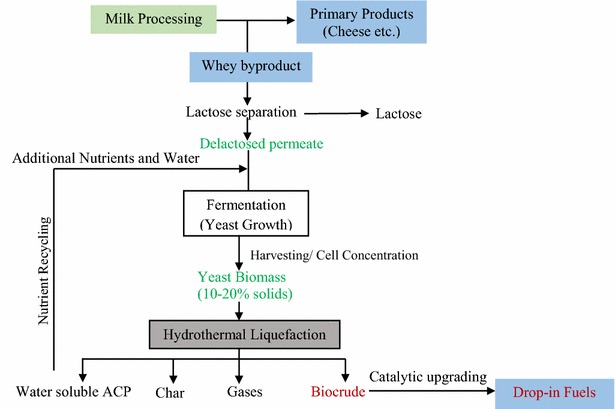
Conceptual oleaginous yeast platform for producing biofuels and improving dairy value-chain. *ACP* aqueous co-phase product)

Yeast is capable of growing and accumulating oil using a wide variety of carbon sources from processing wastes/byproducts including molasses, whey, raw materials from the food industry, lignocellulosic hyrolysates (starch hydrolysate, banana juice, tomato waste hydrolysate, sweet sorghum extracts, wastewater sewage sludge, etc.), glycerol, and hydrophobic materials (vegetable oils, industrial fats, etc.) [1, 47]. Currently, delactosed permeate is a low-value product in the dairy value-chain [48]. Being a rich source of nutrients, this byproduct offers high-value economic opportunities through upgrading with yeast fermentation followed by HTL conversion to biocrude. Based on the present results from the HTL of yeast grown on simulated delactosed permeate medium, a conceptual yeast HTL platform is proposed for high-value utilization of delactosed permeate (Fig. 6). The proposed platform suggests utilizing delactosed permeate for oleaginous yeast growth via fermentation and its subsequent conversion into biocrude via co-solvent HTL. Based on our experimental HTL results an approximate mass flow of substrate and products is shown in Additional file 1: Figure S4. Biofuel conversion efficiency in upgrading of HTL biocrude was assumed as 75 % [9]. The other assumptions in upgrading are as follows: 14 % coke yield, 10 % yield of process gases; H_2_ consumption at 0.035 kg (kg HTL biocrude)^−1^ and catalyst input at 0.004 kg (kg HTL biocrude)^−1^ [49]. Biocrude obtained by HTL can be further upgraded into a hydrocarbon fuel via catalytic hydrogenation and other thermal methods [37, 38, 50]. The soluble organics in the aqueous co-phase products (ACP) have high TOC and can be converted into methane-rich gas [46] that can serve as a hydrogen source for hydrogenation reactions. ACP products from HTL also contain significant amounts of dissolved N and P nutrients. Recycling of nutrients from HTL processing of algae has been suggested by many researchers around the world [21, 42–44]. LCA studies by Frank et al. [51] suggest that reuse of N and P will play a significant role in the HTL process cost economics and scale-up. In our proposed biorefinery platform, N and P in HTL-ACP is recycled for further growth of yeast biomass (Fig. 6). Solid char can be further used as an energy-dense fuel for process heating or other energy uses. Development of fuels and added nutrients in the proposed oleaginous yeast HTL platform can improve the existing dairy value-chain.

## Conclusions

The feasibility of yeast biomass conversion into energy-dense biocrude in a low-temperature co-solvent HTL process was demonstrated using a water–isopropanol binary solvent. HTL of oleaginous yeast, *C. curvatus* produced biocrude at a yield of 48.8–58.9 %. This biocrude had an energy content of 37 MJ kg^−1^ (compared to 42 MJ kg^−1^ for petroleum crude or 39 MJ kg^−1^ for biodiesel). The biocrude was characterized by low O (12–14 %), low N (0.5–2.0 % N), and the presence of C_17_–C_20_ alkanes, fatty acids, and glycerides. The aqueous co-phase products (ACP) contained significant N and P that resulted from reaction and subsequent disposition of N and P from the starting biomass. This research also discusses a conceptual yeast HTL biofuel platform for high-value utilization of dairy byproduct, delactosed permeate in generation of biocrude (fuel), added nutrients, and solids. Initial economic evaluation shows that the HTL process can generate a liquid fuel from the yeast biomass at $5.09 gallon^−1^. Suggested use of waste agriculture streams in a bioenergy-based process can increase the productivity and sustainability of rural areas while providing a new feedstock that has significant advantages compared to first-generation biofuel feedstocks.

## Methods

### Raw materials

#### Strain and culture conditions

Yeast strain, *C. curvatus* (ATCC# 20509) was obtained from the American Type Culture Collection (ATCC, Manassas, VA, USA). *C. curvatus* was preserved at −80 °C in yeast extract peptone dextrose (YPD) media [52] with 20 % (% v/v) glycerol and generally cultured on YPD media.

#### Chemicals and reagents

High-performance liquid chromatography grade acetone and dichloromethane (DCM) (99.8 % v/v) were purchased from Honeywell Burdick & Jackson^®^ (USA) and Cambridge Isotope Laboratories (USA), respectively. Nitrogen and helium gases were obtained from Airgas Inc. (USA). The catalyst, Na_2_CO_3_ was purchased from J.T. Baker Inc. in the anhydrous powder form. Deuterated chloroform (CDCl_3_) (Cambridge Isotope Laboratories Inc, USA) and NMR tubes (Wilmad-Lab Glass, USA) of 5 mm diameter were purchased from the ChemStore, University of Nevada Reno, NV.

### Yeast growth

Yeast growth was performed in a 50-L fermenter with an aeration rate of 3 standard cubic feet per minute at a temperature of 30 °C using a medium described previously, except that lactose was used in place of glucose to simulate delactosed permeate [48, 53]. The fermenter agitator consisted of 3 marine blades rotating at 225 rpm. Culture was inoculated with an overnight culture equal to 5 % of the fermenter volume and allowed to grow for 5 days. Cells were collected by centrifugation, frozen, and then lyophilized.

### Hydrothermal liquefaction and product separation

**Fig. 7:**
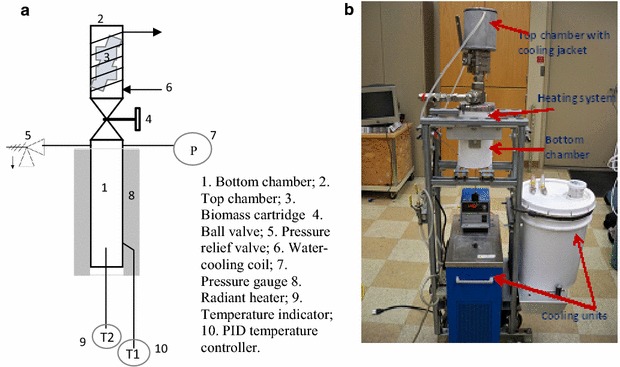
The 2-chamber reactor system used for co-solvent hydrothermal reaction experiments. **a** Schematic of the experimental system, **b** photo of the actual laboratory system

HTL of yeast biomass, *C. curvatus* was performed with or without a catalyst and co-solvent at 240–300 °C and 3.5–8.2 MPa (450–1200 psi) corresponding pressure for 30 min using Desert Research Institute’s 2-chamber reactor and 2-L high pressure reactor systems. The 2-chamber reactor system (Fig. 7) consists of two chambers separated by a ball valve: the bottom chamber holds the desired amount of solvent (water or co-solvent mixture), which is heated to the preset reaction temperature; the top chamber holds the biomass sample, which is dropped into the bottom chamber. The 2-chamber reactor provides excellent control of both temperature and reaction time and has the advantage of quick cooling [54]. However, a limitation is this reactor’s inability to conduct HTL at temperatures and pressures over 280 °C, and 5.1 MPa (750 psi), respectively. Hence, all experiments conducted at 300 °C were performed using a 2-L reactor system (Parr Instruments Co., IL, USA), which was designed to operate at maximum temperature and pressures of 320 °C and 8.2 MPa (1200 psi), respectively. Helium (He) gas was purged thoroughly before heating the reactor systems. The total volume of the 2-chamber reactor is approximately 200 mL. In a typical experiment, 4 g of feedstock and 40 mL of solvent were used, leaving a headspace of about 160 mL. The total volume of the Parr reactor is approximately 2000 mL. In a typical run 40 g of feedstock and 400 mL of solvent were used leaving a headspace of about 1600 mL.

**Fig. 8:**
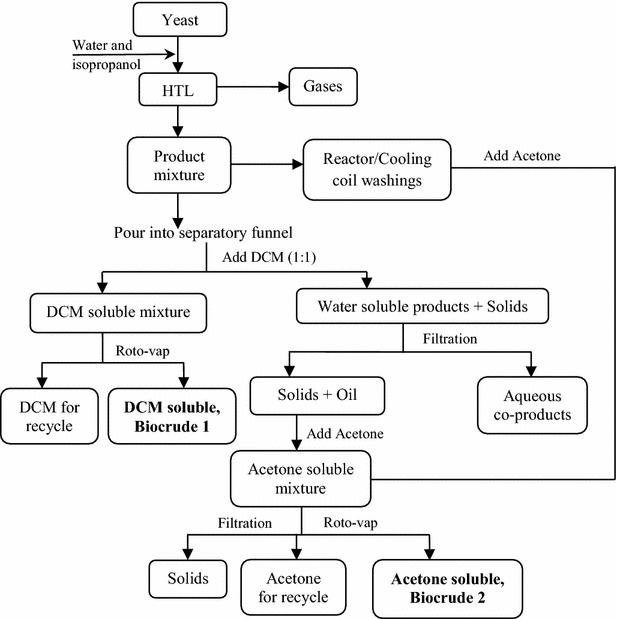
HTL product separation protocol using dichloromethane (DCM) and acetone

The Parr reactor system has been described previously [55]. It includes a stirred-type batch reactor along with a gas sampling system (Additional file 1: Figure S5) and can process ~25 times more dry biomass than the 2-chamber reactor. Reaction temperatures in each reactor system were controlled with a National Instruments LabView data acquisition program utilizing two thermocouples—one installed at the outer wall of the reactor; the other inside the reactor. HTL experiments were typically performed using a 1:10 ratio of biomass to solvent (water or water + isopropanol, 1:1) for 30 min residence time with 300 rpm stirring speed. Na_2_CO_3_ (5 % w/w) was used in all catalytic runs. It is important to note that in the 2-chamber reactor, the sample was exposed to the desired reaction conditions for exactly 30 min, whereas in the 2-L Parr reactor system, the residence time was defined as the holding time once the reaction temperature reached the experimental set point (300 °C in the example shown in Additional file 1: Figure S6). Hence the actual exposure time was longer than 30 min in the Parr reactor. The average calculated heating rate was ~3 °C min^−1^ in the Parr reactor. Corresponding working pressures of the 2-chamber reactor and 2-L Parr reactor were ~3.0 MPa (440 ± 50 psi) at 240 °C and ~8.5 MPa (1235 ± 20 psi) at 300 °C, respectively. The products after a typical HTL experimental run were separated using dichloromethane (DCM) and acetone solvents as shown in Fig. 8. DCM was selected because it is a dense, relatively non-polar, volatile solvent. It is miscible with many organic solvents and can maximize the solubilization of compounds in biocrude obtained from HTL experiment. Also, because DCM is immiscible with water, the aqueous phase can be easily separated by decantation/gravity (Fig. 8).

Product yield was determined as the ratio of mass yield of products to the initial dry weight of yeast biomass used in a particular HTL experiment. Total biocrude yield reported in this study was the sum of the yields of biocrude 1 (B1) and biocrude 2 (B2) that were obtained from the DCM-assisted and acetone-assisted separations, respectively. The different treatments in this study were: (1) non-catalytic HTL at 300 °C, without co-solvent; (2) catalytic HTL at 300 °C, without co-solvent; (3) non-catalytic HTL at 240 °C, with co-solvent, and (4) catalytic HTL at 240 °C, with co-solvent. All HTL runs were performed in duplicate and measurements were performed in triplicate unless otherwise indicated.

### Analytical work

#### Analysis of *C. curvatus* biomass

##### Total protein, lipid, and carbohydrate analysis

Total protein was determined at Midwest Laboratories (Omaha, NE, USA). The amount of nitrogen was determined via combustion and total protein was calculated by multiplying the nitrogen value by 6.25 [56, 57]. Lipids were quantified based on an acid-catalyzed fatty acid methyl ester (FAME) method developed by Wahlen et al. [58]. Samples for FAME analysis were further diluted 1:10 with chloroform in a gas chromatograph (GC) vial such that sample concentrations fell within the detector calibration range. Standards were prepared using pure methyl myristate (C14:0), methyl palmitoleate (C16:1), and methyl oleate (C18:1) (Nu-Chek Prep, Inc., Elysian, MN). FAME mixtures were prepared at final concentrations of 0.1, 0.2, 0.4, 0.6, 0.8, and 1.0 mg mL^−1^ to calibrate the flame ionization detector (FID). FAME content of the sample was determined by GC with a 123-BD11 column using chromatographic conditions identical to those for triglyceride quantitation [58]. Total carbohydrate was determined using the phenol–sulfuric acid method as described previously [59]. Absorbance was read at 490 nm using a Varian 50Bio UV–Vis Spectrophotometer (Varian Inc., USA). Total carbohydrate was determined from the standard curve and accounted for dilution factors.

##### Proximate analyses

Moisture, volatile matter (VM), ash and fixed carbon (FC) of raw yeast and solid char samples were measured using a LECO TGA-701 proximate analyzer (Mettler Toledo, USA; Model: TGA/DSC1) following the ASTM D 5142 method. A standard coal (Vanguard Solutions, Ashland, KY, USA) with 24.05 % ash, 29.51 % VM, and 46.48 % FC was used for calibration. Each analysis sequence included a blank crucible analyzed twice for: (1) thermal equilibration and (2) for blank subtraction as buoyancy compensation. All samples were first analyzed under pure nitrogen using the temperature protocol of the phase 1 method before proceeding under air with phase 2. The phase 1 method was performed under 70 mL min^−1^ of nitrogen flow through the combined 25 mL min^−1^ of protective gas and 45 mL min^−1^ of purge gas. Initial sample mass was taken at the start of the phase 1 method before heating to 107 ± 3 °C for 30 min, then to the upper temperature value (700 °C for biomass) for 60 min. Mass lost during the two heating events was taken as moisture and volatile matter, respectively. The combined mass of fixed carbon and ash was calculated as the initial mass minus the total lost mass in phase 1. The phase 2 method was conducted under air at the same 70 mL min^−1^ flow. The sample was rapidly heated to the upper temperature (700 °C) and the final mass of the remaining sample was taken as the ash content. Fixed carbon was determined by difference.

##### Ultimate analysis

Untreated yeast feedstock was analyzed for elemental C, H, N, and O using a ThermoElectron Flash EA 1112 Automatic Elemental Analyzer. The complete analysis requires two methods, with two separate injections: one for C, H, and N analysis; the other for O analysis. For C, H, and N determination, the sample is weighed in a tin foil capsule, and then dropped into an oxidation/reduction (FeO and Fe) reactor kept at a temperature of 900–1000 °C. The amount of O_2_ necessary for complete combustion is delivered into the reaction chamber. The exothermic reaction between the sample and O_2_ temporarily raises the temperature to about 1800 °C, which is sufficient to convert both organic and inorganic compounds into elemental gases that are reduced and separated on a GC column using He as the carrier gas. The produced gases (N_2_, CO_2_, and H_2_O) are detected and quantified by a thermal conductivity detector (TCD). For oxygen determination, the sample is weighed into a silver foil capsule, then dropped into a reaction chamber containing a nickel-coated carbon catalyst, held at a temperature slightly above 600 °C. Under these conditions, oxygen is converted to CO, which is routed through a water trap before being passed to the GC column for quantification by the TCD.

#### Analysis of HTL products

##### Ultimate analysis

Biocrude samples were analyzed for elemental C, H, N, and O using a ThermoElectron Flash EA 1112 Automatic Elemental Analyzer, as per the procedure described above. *Higher heating values* (HHV) of biocrude and oven-dried solid (feedstock and char) samples were measured using a bomb calorimeter (Parr 6200, Parr Instruments, USA) at the DRI Bioenergy Laboratory.

##### GC–MS analysis

To each HTL biocrude sample prepared as previously described, 100 μL of a derivitization agent, *N*-methyl-*N*-trimethylsilyltrifluoroacetamide (MSTFA) was added. One μL of each sample was injected into the split/splitless injector set to a split ratio of 1:2. Compounds were separated using an HT5 (5 % phenyl polycarborane-siloxane) GC column (30 m, 0.25 mm ID, and 0.10 μm film thickness, SGE, Austin, TX, USA) and detected using a quadrupole mass spectrometer (GCMS-QP2010S, Shimadzu Scientific, Columbia, MD, USA) set to maintain an interface and ion source temperature of 340 and 200 °C, respectively. Helium was used as the carrier gas set to a constant velocity of 50 cm s^−1^. The injector temperature was set at 350 °C and the column was initially set at a temperature of 60 °C for 1 min and then increased to a temperature of 200 °C at a rate of 5 °C min^−1^. The temperature was then raised to 340 °C at a rate of 15 °C min^−1^ and held at this temperature for 5 min. A mass range of 45 to 500 *m/z* was scanned at a rate of 1000 scans s^−1^. Compounds were identified by comparing the retention times of resolved peaks with those of alkane and fatty acid standards, and by comparing the mass fragment pattern of each resolved peak to the National Institute of Standards and Technology (NIST) 2005 mass spectral library (NIST, Gaithersburg, MD) using the software GC/MS postrun analysis v2.3 (Shimadzu Scientific, Columbia, MD). The percent of each peak, as shown in the additional file, was determined by the percent of the total peak area after subtracting the known mass of triglyceride in the sample.

##### Triglyceride quantitation

Triglycerides (TAG) could not be resolved with the mass spectrometer due to temperature limitations, hence the FID detector was utilized to quantify total TAG concentration in the samples. Samples were separated on an Agilent 123-BD11 column (15 m × 0.32 mm ID with 0.1 μm film thickness) (Agilent, Santa Clara, California). Analysis was conducted by injecting 1 μL of the sample into the programmed temperature vaporizing (PTV) inlet, which followed a temperature program of 60 °C for 1 min, followed by a gradient of 10 °C min^−1^ up to 370 °C, then held at this temperature for 6 min. The oven followed an identical temperature program. The FID detector was maintained at a temperature of 380 °C. Helium was used as the carrier gas and the flow was controlled in constant velocity mode at 30 cm s^−1^. The FID detector was calibrated using tripalmitin (Nu-chek Prep Inc., Elysian, MN, USA) at six concentrations ranging from 0.065 to 0.65 mg mL^−1^. Data collected for standards utilized the same GC program. The TAG peaks eluting from 27.5 min to 32.5 min were integrated using GC Solution Postrun v. 2.3 (Shimdzu Scientific, Columbia, MD, USA) and the amount of TAG in each sample was determined by linear regression.

##### Nuclear magnetic resonance spectroscopy (NMR)

^1^H NMR spectra of biocrude samples were recorded at 25 °C using a 2-channel 500 MHz Varian VNMRS with an automation probe at the indicated frequency and referenced to tetramethyl silane (TMS). Samples (7.5 % w/w) were prepared by dissolving 20–25 mg of biocrude oil in deuterated solvent (CDCl_3_) containing 0.03 % TMS as an internal reference and filtering the mixture through a 0.45-µ PTFE filter to remove any suspended particulates before loading into 5-mm diameter NMR tubes.

##### Fourier transform infrared (FT-IR)

FT-IR spectra of yeast raw material and biocrudes were recorded on a Thermo Nicolet 6700 FT-IR spectrometer (Thermoscientific, USA) to determine their functional groups. All samples were analyzed in the wave number range of 4500–600 cm^−1^.

##### Analysis of gases

The non-condensable gases from 2-L Parr reactor runs were collected in a Tedlar bag and analyzed using an SRI 8610C GC equipped with a thermal conductivity detector (TCD) using a method that allowed for measurement of H_2_, CO, CO_2_, and C_1_–C_3_ hydrocarbons in a single analysis [60]. Helium was used as carrier gas, with a flow rate of 11.0 mL min^−1^. Two packed GC columns were utilized: (1) 6-ft × 1/8 in. Molecular Sieve 13X placed in the instrument’s valve oven and maintained at a constant temperature of 90 °C, and (2) 6-ft × 1/8 in. Silica Gel placed in the instrument’s column oven where the temperature could be programmed. An initial column temperature of 40 °C was maintained for 9.0 min., followed by an increase to 200 °C at the rate of 15 °C min^−1^.

##### Analyses of water-soluble aqueous phase co-products (ACP)

ACP obtained from different HTL experiments were filtered with a 0.45 µ Whatman filter before N and P analyses. Measurements of ammonia nitrogen, nitrate nitrogen, total Kjeldahl nitrogen (KN), and total phosphorous (TP) of ACP were performed using Standard USEPA Test Methods [61]. The pH and non-volatile residue (NVR) content of the ACP were measured immediately following completion of the experiments and separation of the products. The pH of the ACP was measured using a portable Hannah Instruments HI 8424 digital pH and temperature meter. The NVR content was measured by weighing triplicate samples of the ACP into drying tins which were then placed in a convective oven at 105 °C overnight (approximately, 18–20 h) to obtain an oven-dried weight. The remaining residue represented the NVR content of the ACP. Total organic carbon (TOC) was measured using a Shimadzu TOC-VCSH instrument (Columbia, MD) that catalytically oxidizes all organic compounds into CO_2_, which is then measured by nondispersive infrared detection (NDIR) [55].

##### SEM analyses of solid char

Scanning electron microscopy (SEM) analyses of unprocessed (raw) yeast and solid char were performed at the Electron Microscopy and Microanalysis Facility, the University of Nevada, Reno, using a FE-SEM Hitachi Scanning Electron Microscope (model S-4700). The SEM samples were prepared by depositing about 50 mg of sample on an aluminum stud covered with conductive adhesive carbon tape, and then coating with a thin layer of gold using a sputter coater (EMI Tech K575X) at 2500 psi Ar gas and 80–90 mA current to prevent charging during observations. Imaging was done in the vacuum mode at an accelerating voltage of 20 kV, using secondary electrons.

### Techno-economic evaluation

**Fig. 9:**
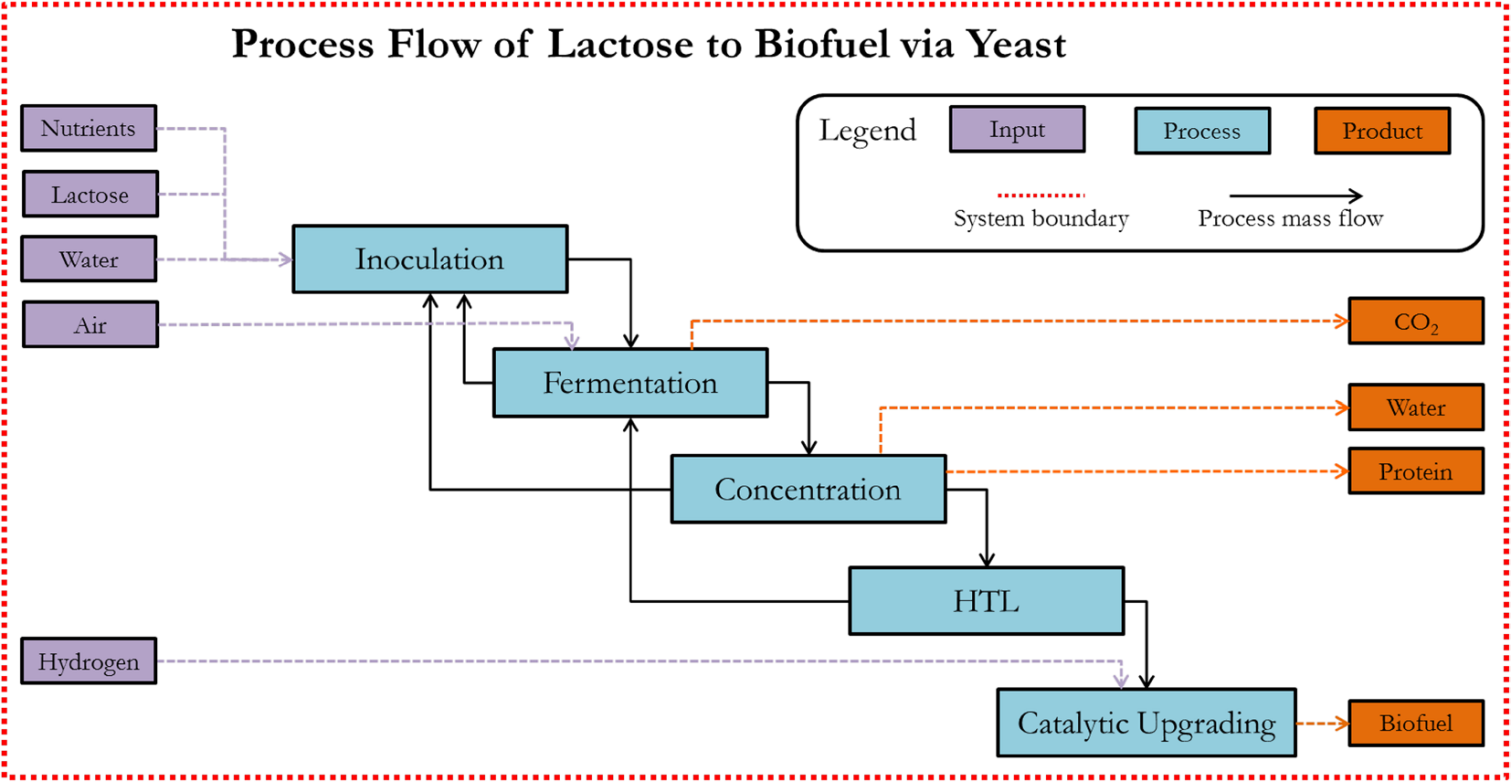
Engineering system model for conversion of lactose to biofuel via yeast

The focus of the economic work is to understand the feasibility of producing biofuel from yeast integrating a thermochemical conversion system and provide data feedback to experimental work such that the economic viability of the process can be improved. Modeling methods are based on the work of Summers et al. [30]. In general, process models were developed to reflect capital and operational costs associated with each sub-process in the delactosed permeate to biofuel conversion pathway based on the baseline HTL conversion process (without catalyst or co-solvent) at a production level of 72 million liters of biofuel per year (Fig. 9). The total capital investment of $94.2 million is required with 33, 4, 49, and 14 % attributed to fermentation, harvesting, HTL, and bio-oil processing and upgrading, respectively. The capital build out is assumed to occur over a 3-year period with 8, 60, and 32 % of the capital spent in year 1, 2, and 3, respectively. Experimental data were leveraged for sub-process model performance and integrated into an engineering system model of the process. Operational costs were determined through the engineering modeling work which was focused on tracking energy consumption and mass balance. The total annual operational cost for the facility excluding labor is $38.4 million with 52, 1, 41, and 8 % attributed to fermentation, harvesting, HTL, and bio-oil processing and upgrading, respectively. Demonstrated upper limits of biomass and lipid productivity of 34.21 g L^−1^ and 40 % lipid of cellular dry weight, respectively, were chosen as inputs to the process models resulting in a biomass production cost of $1205 tonne^−1^, which is economically similar to the fermentation intensive processes of brewing beer [62]. This upstream fermentation cost was used as a fixed input to the downstream process models of the conversion technology presented in this study in an effort to solely investigate cost sensitivity of HTL process inputs. Systems engineering process models reflecting the HTL conversion and upgrading to biofuel were developed using experimental results obtained from literature [9].

Process models within the system boundary were combined to generate an engineering system model that was further leveraged to perform a techno-economic analysis to determine commercial feasibility of the biofuel production pathway. Capital and operational costs for each process were selected to reflect an industrial-scale conversion plant. Economic assumptions include a 10 % internal rate of return, 35 % income tax rate and a 30-year plant life, were based on the Department of Energy Bioenergy Technologies Office (BETO) standards to provide a means for comparing various proposed biofuel plants and adapted from Summers et al. [30]. Further details on the economic and operational assumptions are presented in the Additional file 1: Tables S3 and S4.

### Methods of data processing

#### Material and energy recovery (ER)

Mass recovery (i.e., yield) of a particular product fraction was defined as the ratio of mass yield of the product (biocrude, char, gas, or NVR) to the dry mass of the starting yeast feedstock and was expressed in percentage. One-way ANOVA was performed using IBM SPSS 20.0 statistical package to report the difference in mass yields of biocrudes (at a significance level, *p* = 0.05). The energy recovery in biocrude (ER_biocrude_) was calculated from the yield data and expressed as percentage of the total chemical energy recovered in biocrude as [29]:

5 $$ {\text{ERbiocrude}} = \frac{{{\text{HHV of biocrude }} \times {\text{mass of biocrude}}}}{{{\text{HHV of raw feedstock }} \times {\text{mass of raw feedstock}}}}\, \times\, 1 0 0 {\text{\% }} $$

## Additional file

10.1186/s13068-015-0345-5 Supplementary HTL data and experimental information.

## Abbreviations

C: carbon
H: hydrogen
N: nitrogen
O: oxygen
H_2_: hydrogen
CO: carbon monoxide
CO_2_: carbon dioxide
GC: gas chromatography
KN: Kjeldahl nitrogen
MS: mass spectrometry
TP: total phosphorous
ACP: aqueous co-products
DCM: dichloromethane
HHV: higher heating value
HTL: hydrothermal liquefaction
NMR: nuclear magnetic resonance spectroscopy
TOC: total organic carbon
TMS: tetramethyl silane
ATCC: American Type Culture Collection
CSHTL: co-solvent hydrothermal liquefaction
FAME: fatty acid methyl esters
