# Supplementary material for: Oleaginous yeast platform for producing biofuels via co-solvent hydrothermal liquefaction
Source: Biotechnol Biofuels. 2015 Oct 13;8:167. doi: 10.1186/s13068-015-0345-5 (PMC4605089; doi:10.1186/s13068-015-0345-5)
Supplement: Supplementary file 1 — 10.1186/s13068-015-0345-5 Supplementary HTL data and experimental information. [file 13068_2015_345_MOESM1_ESM.docx]

Supplementary Information for

**Oleaginous Yeast Platform for Producing Biofuels via Co-Solvent Hydrothermal Liquefaction**

Umakanta Jena^†1^, Alex T. McCurdy^2^, Andrew Warren^1^, Hailey Summers^2^, Rhesa N. Ledbetter^2^, S. Kent Hoekman^1^, Lance C. Seefeldt^2^, Jason C. Quinn^2^

^1^Desert Research Institute, Reno, NV-89512; ^2^Utah State University, Logan, UT- 84322

^†^*Corresponding Author:*

Email: [Umakanta.Jena@dri.edu](mailto:Umakanta.Jena@dri.edu); Ph: +1 775-674-7122; Fax: +1 775-674-7016

**Figure S-1**. Biocrude yield in different HTL treatment runs (for 30 min residence time).

|  |
| --- |


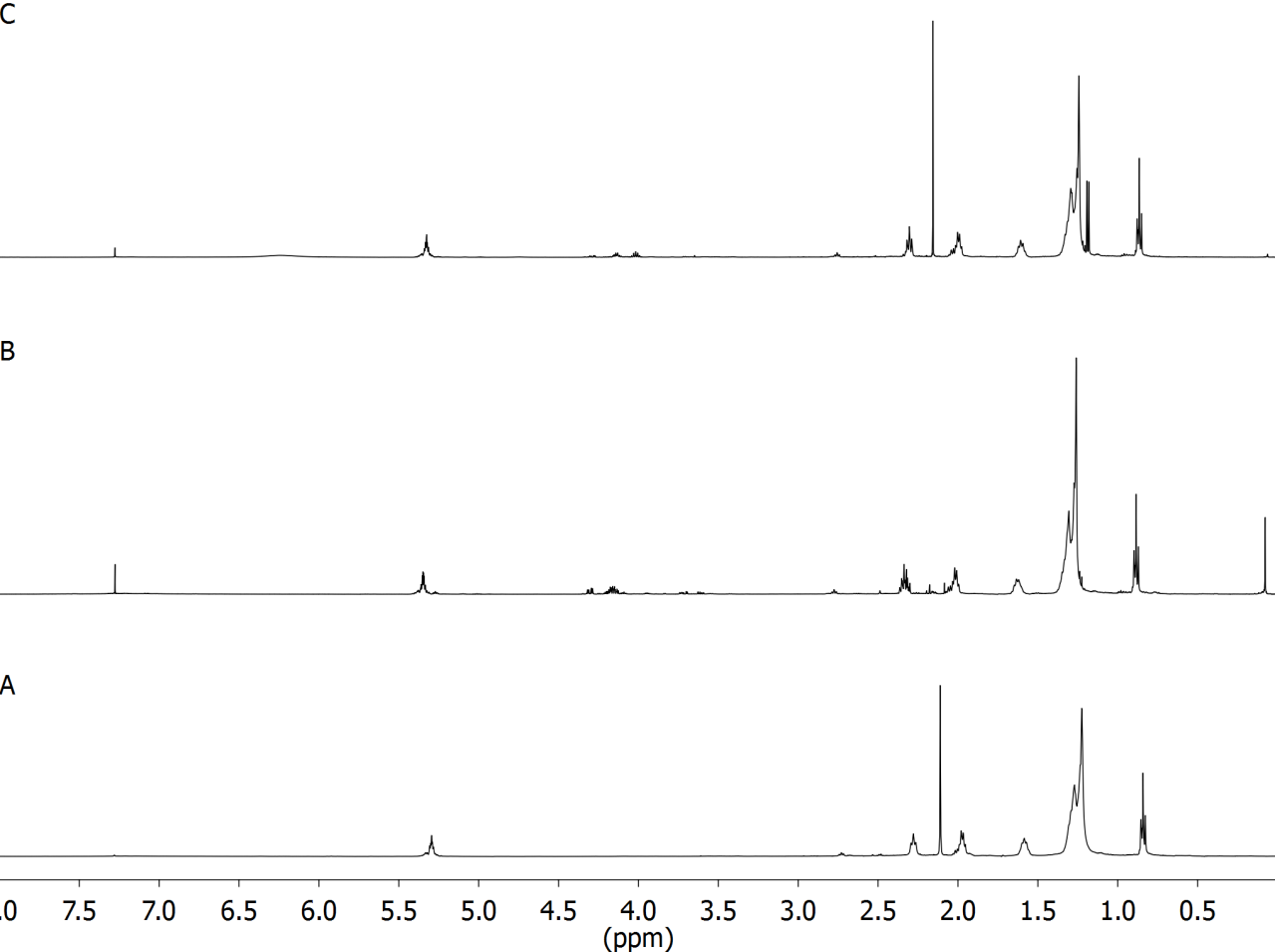


**Figure S-2.** ^1^H NMR spectra (500 MHz, 25 °C, CDCl_3_) of biocrude from: (A) HTL run, 2-L Parr reactor, at 300 °C, w/o a co-solvent, with Na_2_CO_3_ catalyst, (B) non-catalytic HTL runs, 2-chamber reactor, 240 °C, with co-solvent, (C) catalytic HTL runs, 2-chamber reactor, 240 °C, with co-solvent. (The sharp peak seen near 2.1 ppm is due to acetone.)


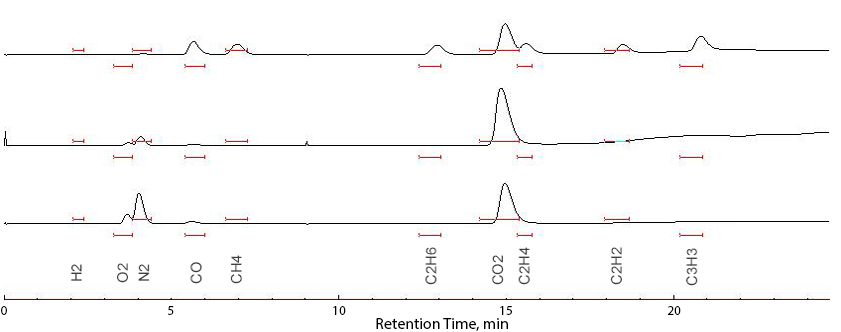


B

C

A

**Figure S-3**. Gas chromatograms of (A) catalytic HTL run at 300 °C and (B) non-catalytic HTL run at 300 °C, and (C) calibration standard.


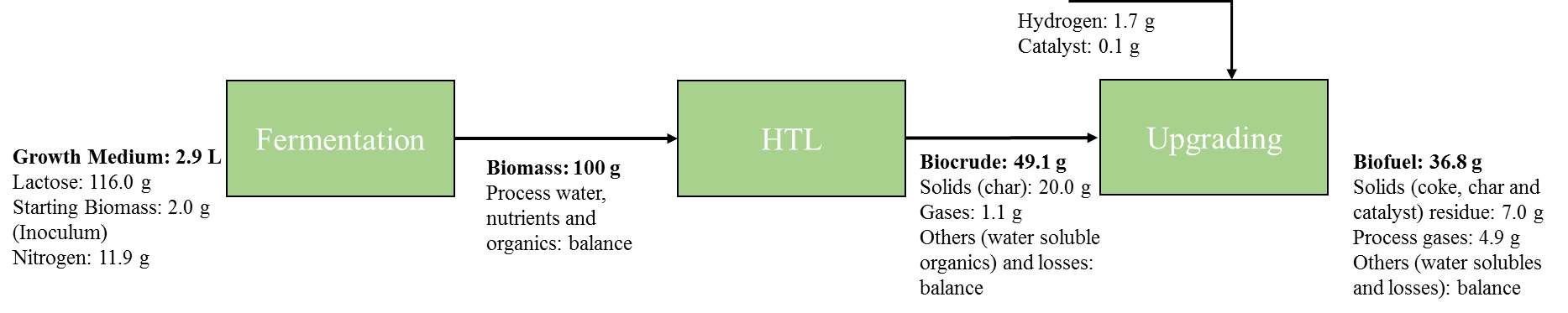


Figure S-4: Schematic diagram showing mass flow of substrate and products in the proposed yeast to biofuel system. Mass was calculated for 100 g unit yeast biomass (dry) basis; *Assumptions for upgrading: biocrude conversion into biofuel @75% [9]; coke yield @20%; process gases @10%; H_2_ consumption @0.035 kg/kg HTL biocrude; catalyst input @0.004 kg/kg HTL biocrude [50].*

| 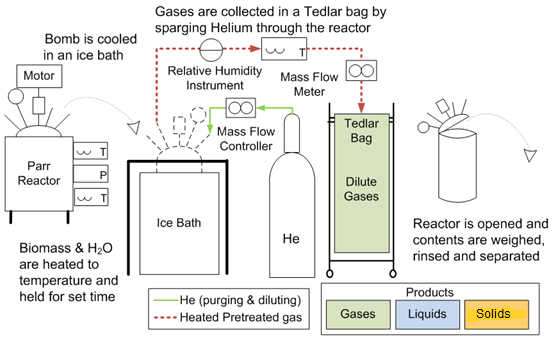  (a) | 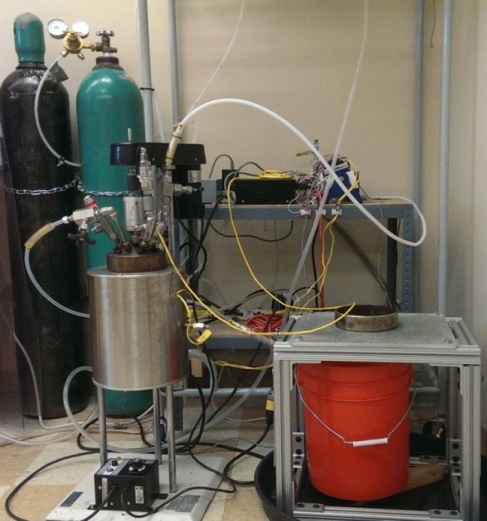  Ice-cooling bucket  (b) |
| --- | --- |

**Figure S-5.** a) Schematic of the hydrothermal liquefaction experiment conducted in a 2-L Parr reactor, and b) Photograph showing the reactor set-up with external cooling arrangement.


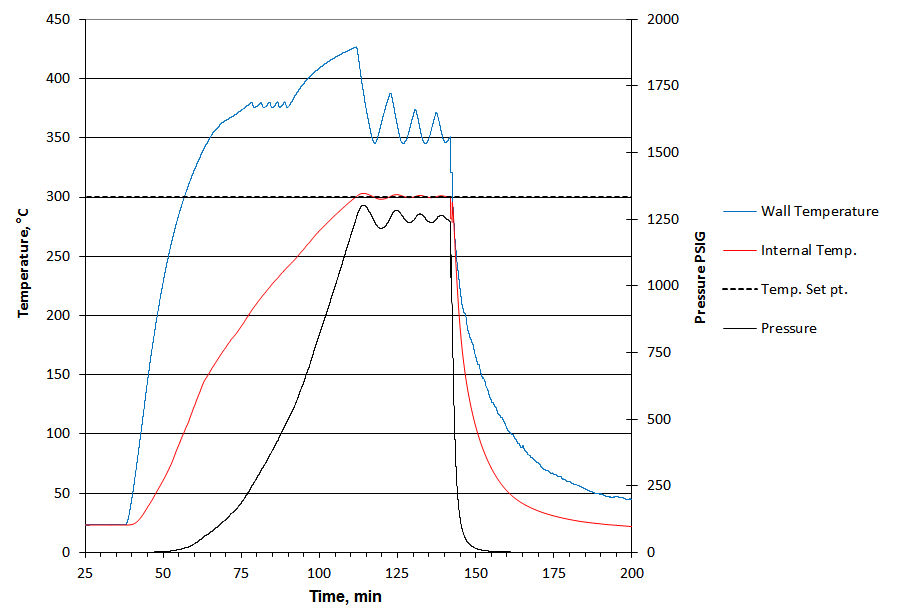


A


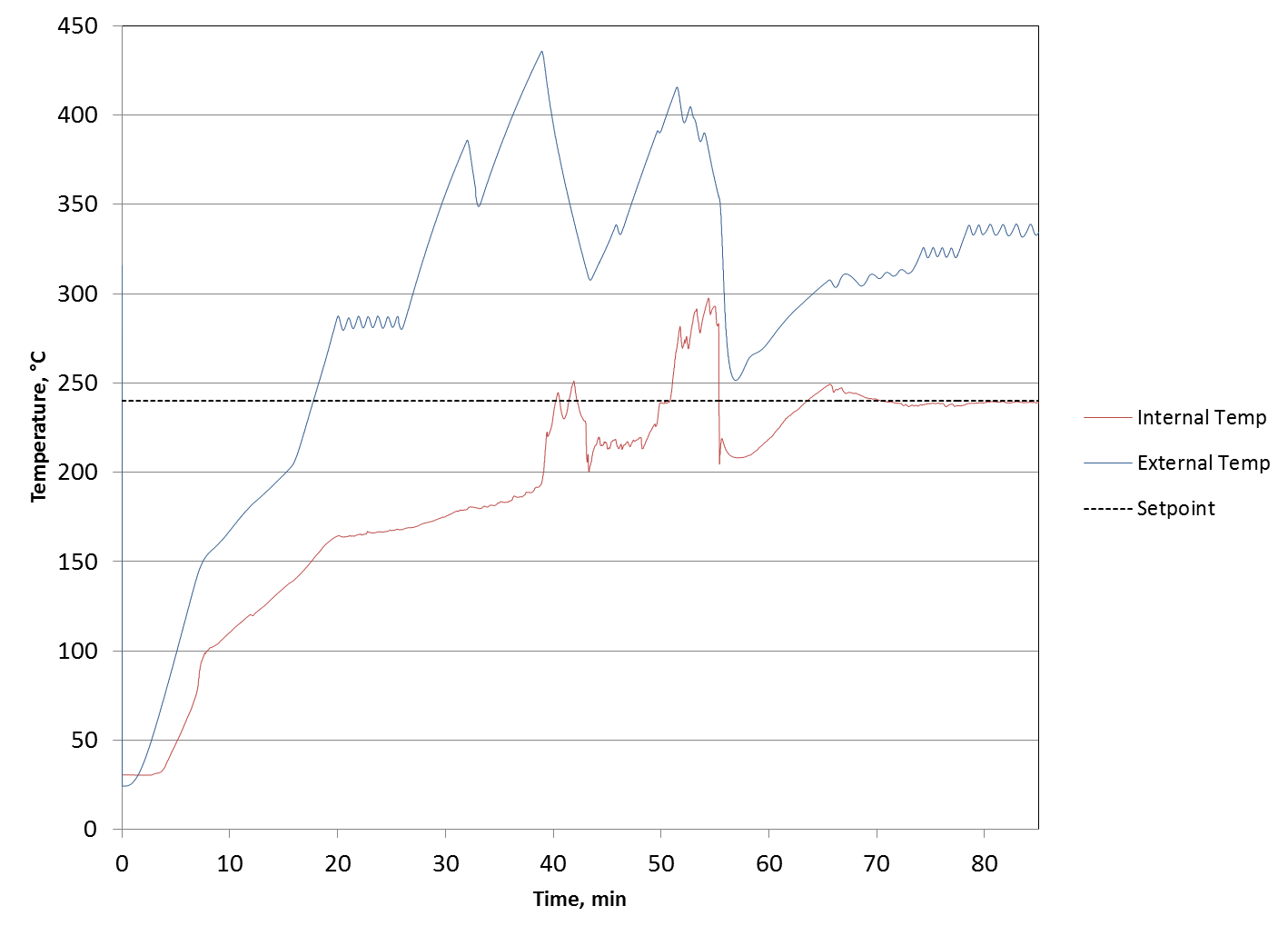


B

**Figure S-6**. Temperature and pressure profiles of typical runs in (A) 2-L Parr Reactor, and (B) Two-chamber reactor systems. Corresponding working pressures of the 2-L Parr reactor and 2-chamber reactors were 1235±20 psi (300 °C) and 440±50 psi (240 °C), respectively. For the 2-chamber reactor the reported pressure was a calculated parameter.

Table S-1. GC-MS Identification of compounds in biocrude obtained from the DCM extracted (B1) and acetone extracted (B2) biocrudes at different HTL experimental conditions

| Compounds | Retention time, min | Relative Abundance, % | | | | | | | |
| --- | --- | --- | --- | --- | --- | --- | --- | --- | --- |
|  |  | Non-catalytic HTL w/o co-solvent, 300 °C, 2-L | | Catalytic HTL, w/o co-solvent, 300 °C, 2-L | | Co-solvent HTL, non-catalytic, 240 °C, 2-C | | Co-solvent HTL, non-catalytic, 240 °C, 2-C | |
|  |  | B1 | B2 | B1 | B1 | B1 | B2 | B1 | B2 |
| Glycerol (TMSE) | 9.81 | 0.11 | 0.12 | 0.12 | 1.48 | 0.12 | 1.54 | 0.11 | 9.33 |
| Heptadecane | 20.51 | 0.30 | 0.40 | 0.40 | 0.36 | 0.19 | 1.99 | 0.18 | 1.73 |
| D-arabino-hexanoic acid-3-deoxy-2,5,6-tris-gamma-lactone (TMSE) | 23.30 | - | - | - | - | - | 0.29 | - | 8.27 |
| Palmitic acid (Non-deriv.) | 26.30 | 1.20 | 1.28 | 1.28 | 0.76 | - | - | 0.10 | - |
| Eicosane | 26.75 | 0.77 | 1.12 | 1.12 | 0.91 | 0.58 | 1.22 | 0.50 | 1.40 |
| Palmitic acid (TMSE) | 27.82 | 16.85 | 17.32 | 17.32 | 17.78 | 3.51 | 4.57 | 2.43 | 1.19 |
| Oleic acid (Non-deriv.) | 29.74 | 16.12 | 26.82 | 26.82 | 23.46 | 1.81 | 2.52 | 1.90 | 2.31 |
| Stearic acid (Non-deriv.) | 30.19 | 3.54 | 3.37 | - | 2.88 | 0.98 | 1.36 | 1.29 | 2.26 |
| Oleic acid (TMSE) | 30.50 | 46.51 | 40.48 | 40.48 | 42.10 | 6.65 | 6.90 | 5.63 | 1.84 |
| Stearic acid (TMSE) | 30.78 | 13.77 | 10.39 | 10.39 | 8.99 | 2.59 | 2.43 | 1.91 | 1.41 |
| Hexadecenamide | 32.50 | 0.60 | 0.89 | 0.89 | 0.60 | - | - | - | - |
| Octadecenamide | 32.72 | - | 0.23 | 0.23 | - | - | - | - | - |
| Monoglycerides (TMSE) | 33.39 | - | - | - | - | 2.44 | 1.95 | 2.28 | 0.87 |
| Diglycerides (TMSE) | 34.88 | - | 0.29 | 0.29 | - | 8.76 | 19.63 | 8.17 | 14.27 |
| Triglycerides | N/A | - | 0.66 | 0.66 | 0.66 | 72.37 | 55.60 | 75.50 | 55.12 |

*B1: Biocrude1, B2: Biocrude2; DCME: Dichloromethane extracted; AE: Acetone extracted; NA: not available; TMSE: Trimethylsilyl ester and Trimethyl ether: 2-L: for Parr reactor, 2-C: for 2-chamber reactor*

Table S-2. Yields and composition of solid char samples obtained from HTL of yeast (all values are from the average of three measurements)

|  | Non-catalytic HTL, w/o co-solvent, | Catalytic HTL^a^, w/o co-solvent | Co-solvent HTL^b^, non-catalytic | Co-solvent HTL, catalytic^a^ |
| --- | --- | --- | --- | --- |
| Temperature/reactor type | 300 ^o^C, 2-L | 300 °C, 2-L | 240 °C, 2-Chamber | 240 °C, 2-Chamber |
| *Proximate analysis* |  |  |  |  |
| Moisture, % | 6.13 | nd | 1.56 | nd |
| V.M., % | 87.83 | nd | 84.57 | nd |
| F.C., % | 8.24 | nd | 8.57 | nd |
| Ash, % | 3.93 | nd | 6.86 | nd |
| *Elemental composition* |  |  |  |  |
| C, % | 69.53 | nd | 68.11 | nd |
| H, % | 9.36 | nd | 10.77 | nd |
| N, % | 1.51 | nd | 1.66 | nd |
| O, % (by difference) | 19.60 | nd | 19.45 | nd |
| HHV, MJ kg^-1^ | 30.69 | 27.93 | 27.99 | 27.96 |
| Process Chemical Energy Balance, 100 kg yeast input | | | | |
| *Energy In* |  |  |  |  |
| Feedstock Energy, MJ | 2488 | 2488 | 2488 | 2488 |
| *Energy Out* |  |  |  |  |
| Biocrude, MJ | 1784 | 1967 | 2068 | 2180 |
| Solid (char), MJ | 616 | 596 | 858 | 918 |
| *Energy Out* | 2400 | 2563 | 2926 | 3098 |

*HHV:* *Higher heating value; nd: not determined; All HTL runs were performed for 30 min residence time;* ^a^*Catalyst was Na_2_CO_3_ (5% (w/w) of feedstock); ^b^Co-solvent was isopropanol (1:1, in water),*

Table S-3 Foundational economic inputs for techno-economic assessment.

| **Economic Inputs** | |
| --- | --- |
| Plant Operational Days Per Year | 329 |
| Electricity ($/kWh) | 0.07 |
| Natural Gas ($/MMBtu) | 4.25 |
| Equity | 60% |
| Investment Capital | 40% |
| Loan Interest | 15% |
| Loan Term (Years) | 10 |
| Internal Rate of Return | 10% |
| Income Tax Rate | 35% |
| **Construction Period** | |
| %Spent in Year -2 | 8% |
| %Spent in Year -1 | 60% |
| %Spent in Year 0 | 32% |
| **Start Up Time** | |
| Production % of Normal (Year 1) | 50% |
| Fixed OpX % | 100% |

Table S-4 Foundational biological inputs for techno-economic assessment. Values shown represent yields for non-catalytic HTL w/o co-solvent.

| **Model Parameter** |  | **unit** |
| --- | --- | --- |
| Waste Delactosed Permeate | 3.78*10^6^ | L d^-1^ |
| Lactose Density | 120 | g L^-1^ |
| Ammonium Sulfate | 5.0 | g L^-1^ |
| Lactose Concentration for Fermentation | 30 | g L^-1^ |
| Yeast Inoculation Density | 1.62 | g L^-1^ |
| Yeast Harvest Density | 34.2 | g L^-1^ |
| Mass Lost in Centrifugation | 5.0 | % |
| HTL Biomass Conversion Efficiency | 49.11 | % |
| Biocrude Processing Efficiency (hydrocracking & hydrotreating) | 46.9 | % |
